# Tuberculosis Screening, Testing, and Treatment of U.S. Health Care Personnel: Recommendations from the National Tuberculosis Controllers Association and CDC, 2019

**DOI:** 10.15585/mmwr.mm6819a3

**Published:** 2019-05-17

**Authors:** Lynn E. Sosa, Gibril J. Njie, Mark N. Lobato, Sapna Bamrah Morris, William Buchta, Megan L. Casey, Neela D. Goswami, MaryAnn Gruden, Bobbi Jo Hurst, Amera R. Khan, David T. Kuhar, David M. Lewinsohn, Trini A. Mathew, Gerald H. Mazurek, Randall Reves, Lisa Paulos, Wendy Thanassi, Lorna Will, Robert Belknap

**Affiliations:** ^1^Connecticut Department of Public Health; ^2^National Tuberculosis Controllers Association, Smyrna, Georgia; ^3^Division of Tuberculosis Elimination, National Center for HIV/AIDS, Viral Hepatitis, STD, and TB Prevention, CDC; ^4^Logistics Health Incorporated, La Crosse, Wisconsin; ^5^American College of Occupational and Environmental Medicine, Elk Grove Village, Illinois; ^6^Respiratory Health Division, National Institute for Occupational Safety and Health, CDC; ^7^Association of Occupational Health Professionals in Healthcare, Warrendale, Pennsylvania; ^8^Division of Healthcare Quality Promotion, National Center for Emerging and Zoonotic Infectious Diseases, CDC; ^9^Oregon Health & Science University, Portland; ^10^Beaumont Hospital, Royal Oak, Michigan; ^11^Denver Health and Hospital Authority, Denver Public Health, Denver, Colorado; ^12^Maryland Department of Health; ^13^Veterans Administration Palo Alto Healthcare System, Palo Alto, California.

The 2005 CDC guidelines for preventing *Mycobacterium tuberculosis* transmission in health care settings include recommendations for baseline tuberculosis (TB) screening of all U.S. health care personnel and annual testing for health care personnel working in medium-risk settings or settings with potential for ongoing transmission (*1*). Using evidence from a systematic review conducted by a National Tuberculosis Controllers Association (NTCA)-CDC work group, and following methods adapted from the Guide to Community Preventive Services (*2*,*3*), the 2005 CDC recommendations for testing U.S. health care personnel have been updated and now include 1) TB screening with an individual risk assessment and symptom evaluation at baseline (preplacement); 2) TB testing with an interferon-gamma release assay (IGRA) or a tuberculin skin test (TST) for persons without documented prior TB disease or latent TB infection (LTBI); 3) no routine serial TB testing at any interval after baseline in the absence of a known exposure or ongoing transmission; 4) encouragement of treatment for all health care personnel with untreated LTBI, unless treatment is contraindicated; 5) annual symptom screening for health care personnel with untreated LTBI; and 6) annual TB education of all health care personnel.

## Background

Historically, U.S. health care personnel were at increased risk for LTBI and TB disease from occupational exposures; however, recent data suggest that this might no longer be the case. TB rates in the United States have declined substantially; the annual national TB rate in 2017 (2.8 per 100,000 population) represents a 73% decrease from the rate in 1991 (10.4) and a 42% decrease from the rate in 2005 (4.8) (*4*,*5*). Surveillance data reported to CDC during 1995–2007 revealed that TB incidence rates among health care personnel were similar to those in the general population (*6*), raising questions about the cost-effectiveness of routine serial occupational testing (*7*). In addition, a recent retrospective cohort study of approximately 40,000 health care personnel at a tertiary U.S. medical center in a low TB-incidence state found an extremely low rate of TST conversion (0.3%) during 1998–2014, with a limited proportion attributable to occupational exposure (*8*). Moreover, IGRAs and TSTs have well-documented limitations for serial testing of health care personnel at low risk for LTBI and TB disease (*9*,*10*).

## Methods

In 2015, an NTCA-CDC work group comprising experts in TB, infection control, and occupational health was formed to discuss potential updates to recommendations for health care personnel TB screening and testing. The work group included representation from CDC, state and local public health departments, academia, and occupational health associations. During 2015–2016, the work group met periodically to discuss where updates were needed to the 2005 CDC recommendations and to establish a plan for the review of evidence. In January 2017, the work group commenced a systematic literature review of the screening and testing of health care personnel for TB and discussed the findings during a web conference in September 2017. Updated recommendations were developed by the work group during a web conference in December 2017.

**Systematic review methods and findings.** A systematic review of evidence published after release of the 2005 guidelines was conducted using methodology developed for the Guide to Community Preventive Services (*2*,*3*). The search included articles indexed in MEDLINE, EMBASE, and Scopus. The medical subject headings used for the search were “latent tuberculosis” and “tuberculosis”; search terms included “healthcare worker,” “healthcare personnel,” “health worker,” “occupational exposure,” and “occupational diseases.” English language articles were included that 1) were published during January 2006–November 2017; 2) described TB screening and testing in low-incidence (*11*), high-income countries (*12*); 3) employed study designs that were randomized controlled trials, prospective cohort, retrospective cohort, or cross-sectional studies; and 4) reported LTBI prevalence, test conversion or reversion, or TB transmission rates. Each study was independently abstracted and assessed for suitability of study design by two reviewers using a data abstraction form adapted from the Guide to Community Preventive Services (*3*).

This search identified 1,147 citations, of which 39 studies focused on TB screening and testing among health care personnel; three studies (one that was an economic evaluation, one that focused only on test performance, and one of limited execution quality) were excluded, leaving 36 studies in the analysis (Supplementary Box, https://stacks.cdc.gov/view/cdc/77668). Sixteen (44%) of these had been conducted in the United States, with the remaining studies from Australia (one), Europe (17), Israel (one), and New Zealand (one). Thirty-four (94%) studies had been conducted in a hospital setting; most used either a retrospective cohort or cross-sectional design (14). Substantial unexplained heterogeneity existed for all outcomes examined, even when stratified by location or study design. An examination of the patterns of results did not indicate publication bias.

Five U.S. studies reported prior bacillus Calmette-Guérin vaccination by health care personnel (median percentage = 7%; range = 2.3%–93%). Eight of the 16 U.S. studies reported two-step TST testing at baseline. The remaining studies reported IGRA (six) or a combination of IGRA and TST (two) at baseline. Findings from the metaanalyses indicated that 5% and 3% of U.S. health care personnel tested positive at baseline by IGRA and TST, respectively, and that 4% and 0.7% converted from a negative to a positive during serial testing by IGRA and TST, respectively. Among U.S. health care personnel who had a baseline positive test and were retested by the same method during serial testing, the second test was negative in 48% of cases by IGRA and 62% by TST. No U.S. studies were found that evaluated the clinical implications of these discordant results. Among 63,975 U.S. health care personnel from eight studies reporting disease occurrence, none experienced TB disease. Based on expert opinion from the NTCA-CDC work group and findings from the systematic review indicating that a limited proportion of health care personnel test positive at baseline and convert during serial testing, recommendations were drafted for presentation to the Advisory Council on the Elimination of Tuberculosis (ACET) and the Healthcare Infection Control Practices Advisory Committee (HICPAC).

**Expert consultation results.** The draft NTCA-CDC recommendations were presented publicly at the April 2018 ACET meeting (*13*) and the May 2018 HICPAC meeting (*14*). Members of ACET and HICPAC were asked to provide feedback to CDC regarding the recommendations and their accuracy, practicability, clarity, and usefulness. Commenters during the ACET meeting noted that the recommendation encouraging treatment of health care personnel with LTBI could potentially generate cost savings and play an important role in the elimination of active TB disease in the United States. Commenters during the HICPAC meeting were supportive of the need to reduce TB testing for health care personnel; questions were raised about the evidence for, and feasibility of, implementing some of the proposed changes. Commenters during both meetings also encouraged the work group’s plan for a supplemental document to aid health care facilities in implementing the updated recommendations. In addition, the recommendations were presented by NTCA at the National Tuberculosis Conference in May 2018 (*15*) for comment and feedback. Conference attendees supported the need for updated guidelines and the content of the recommendations that were presented.

In July 2018, the NTCA-CDC work group held another web conference to address feedback received from the ACET, HICPAC, and National Tuberculosis Conference meetings and finalized the updated recommendations. The work group requested that NTCA convene a new work group to develop the supplemental implementation guidance document supported by ACET and HICPAC. The supplemental document is expected to be completed by NTCA in 2019.

## Updated Recommendations

Recommendations from the 2005 CDC guidelines that are outside the scope of health care personnel screening, testing, treatment, and education remain unchanged (Table); this includes continuing facility risk assessments for guiding infection control policies and procedures. Here, TB screening is defined as a process that includes a TB risk assessment, symptom evaluation, TB testing for *M. tuberculosis* infection (by either IGRA or TST) for health care personnel without documented evidence of prior LTBI or TB disease, and additional workup for TB disease for health care personnel with positive test results or symptoms compatible with TB disease. This update does not include recommendations for using an IGRA versus a TST for diagnosing LTBI, which have been published elsewhere (*16*).

**TABLE:** Comparison of 2005* and 2019^†^ recommendations for tuberculosis (TB) screening and testing of U.S. health care personnel (HCP)

Category	2005 Recommendation	2019 Recommendation
**Baseline (preplacement) screening and testing**	TB screening of all HCP, including a symptom evaluation and test (IGRA or TST) for those without documented prior TB disease or LTBI.	TB screening of all HCP, including a symptom evaluation and test (IGRA or TST) for those without documented prior TB disease or LTBI **(unchanged)**; individual TB risk assessment **(new)**.
**Postexposure screening and testing**	Symptom evaluation for all HCP when an exposure is recognized. For HCP with a baseline negative TB test and no prior TB disease or LTBI, perform a test (IGRA or TST) when the exposure is identified. If that test is negative, do another test 8–10 weeks after the last exposure.	Symptom evaluation for all HCP when an exposure is recognized. For HCP with a baseline negative TB test and no prior TB disease or LTBI, perform a test (IGRA or TST) when the exposure is identified. If that test is negative, do another test 8–10 weeks after the last exposure **(unchanged)**.
**Serial screening and testing for HCP without LTBI**	According to health care facility and setting risk assessment. Not recommended for HCP working in low-risk health care settings. Recommended for HCP working in medium-risk health care settings and settings with potential ongoing transmission.	Not routinely recommended **(new)**; can consider for selected HCP groups **(unchanged)**; recommend annual TB education for all HCP **(unchanged)**, including information about TB exposure risks for all HCP **(new emphasis)**.
**Evaluation and treatment of positive test results**	Referral to determine whether LTBI treatment is indicated.	Treatment is encouraged for all HCP with untreated LTBI, unless medically contraindicated **(new)**.

**Abbreviations:** IGRA = interferon-gamma release assay; LTBI = latent tuberculosis infection; TST = tuberculin skin test.

* Jensen PA, Lambert LA, Iademarco MF, Ridzon R. Guidelines for preventing the transmission of *Mycobacterium tuberculosis* in health-care settings, 2005. MMWR Recomm Rep 2005;54(No. RR-17). https://www.cdc.gov/mmwr/preview/mmwrhtml/rr5417a1.htm.

**^†^** All other aspects of the Guidelines for Preventing the Transmission of *Mycobacterium tuberculosis* in Health-Care Settings, 2005 remain in effect, including facility risk assessments to help guide infection control policies and procedures.

**Baseline (preplacement) screening and testing.** All U.S. health care personnel should have baseline TB screening, including an individual risk assessment (Box), which is necessary for interpreting any test result. The 2005 guidelines state that baseline test results provide a basis for comparison in the event of a potential or known exposure to *M. tuberculosis*, facilitate detection and treatment of LTBI or TB disease in health care personnel before placement, and reduce the risk to patients and other health care personnel (*1*). The risk assessment and symptom evaluation help guide decisions when interpreting test results. For example, health care personnel with a positive test who are asymptomatic, unlikely to be infected with *M. tuberculosis*, and at low risk for progression on the basis of their risk assessment should have a second test (either an IGRA or a TST) as recommended in the 2017 TB diagnostic guidelines of the American Thoracic Society, Infectious Diseases Society of America, and CDC (*16*). In this example, the health care personnel should be considered infected with *M. tuberculosis* only if both the first and second tests are positive.

BOX Indicators of risk* for tuberculosis (TB) at baseline health care personnel assessment^†^

**Health care personnel should be considered to be at increased risk for TB if they answer “yes” to any of the following statements.**

1. Temporary or permanent residence (for ≥1 month) in a country with a high TB rate (i.e., any country other than Australia, Canada, New Zealand, the United States, and those in western or northern Europe)

Or

2. Current or planned immunosuppression, including human immunodeficiency virus infection, receipt of an organ transplant, treatment with a TNF-alpha antagonist (e.g., infliximab, etanercept, or other), chronic steroids (equivalent of prednisone ≥15 mg/day for ≥1 month), or other immunosuppressive medication

Or

3. Close contact with someone who has had infectious TB disease since the last TB test

**Abbreviation:** TNF = tumor necrosis factor.

* Individual risk assessment information can be useful in interpreting TB test results. (Lewinsohn DM, Leonard MK, LoBue PA, et al. Official American Thoracic Society/Infectious Diseases Society of America/Centers for Disease Control and Prevention clinical practice guidelines: diagnosis of tuberculosis in adults and children. Clin Infec Dis 2017;64:111–5). https://academic.oup.com/cid/article/64/2/111/2811357

**^†^** Adapted from a tuberculosis risk assessment form developed by the California Department of Public Health. https://www.cdph.ca.gov/Programs/CID/DCDC/CDPH%20Document%20Library/TBCB-CA-TB-Risk-Assessment-and-Fact-Sheet.pdf.

**Postexposure screening and testing.** After known exposure to a person with potentially infectious TB disease without use of adequate personal protection, health care personnel should have a timely symptom evaluation and additional testing, if indicated. Those without documented evidence of prior LTBI or TB disease should have an IGRA or a TST performed. Health care personnel with documented prior LTBI or TB disease do not need another test for infection after exposure. These persons should have further evaluation if a concern for TB disease exists. Those with an initial negative test should be retested 8–10 weeks after the last exposure, preferably by using the same test type as was used for the prior negative test.

**Serial screening and testing for health care personnel without LTBI.** In the absence of known exposure or evidence of ongoing TB transmission, U.S. health care personnel (as identified in the 2005 guidelines) without LTBI should not undergo routine serial TB screening or testing at any interval after baseline (e.g., annually). Health care facilities might consider using serial TB screening of certain groups who might be at increased occupational risk for TB exposure (e.g., pulmonologists or respiratory therapists) or in certain settings if transmission has occurred in the past (e.g., emergency departments). Such determinations should be individualized on the basis of factors that might include the number of patients with infectious pulmonary TB who are examined in these areas, whether delays in initiating airborne isolation occurred, or whether prior annual testing has revealed ongoing transmission. Consultation with the local or state health department is encouraged to assist in making these decisions.

Health care personnel might have risks for TB exposure that are not related to their work in the United States, or they might have risks for TB progression after baseline testing that necessitate special consideration. If these risks are unrecognized, these health care personnel might experience TB disease and transmit TB to patients, coworkers, or other contacts. Therefore, health care facilities should educate all health care personnel annually about TB, including risk factors, signs, and symptoms; facilities also should encourage health care personnel to discuss any potential occupational or nonoccupational TB exposure with their primary care provider and occupational health clinician. The decision to perform TB testing after baseline should be based on the person’s risk for TB exposure at work or elsewhere since that person’s last test.

**Evaluation and treatment of health care personnel with positive test results.** Health care personnel with a newly positive test result (with confirmation for those persons at low risk as described previously) should undergo a symptom evaluation and chest radiograph to assess for TB disease. Additional workup might be indicated on the basis of those results. Health care personnel with a prior positive TB test and documented normal chest radiograph do not require a repeat radiograph unless they are symptomatic or starting LTBI treatment (*16*). The local public health department should be notified immediately if TB disease is suspected.

Health care personnel with LTBI and no prior treatment should be offered, and strongly encouraged to complete, treatment with a recommended regimen, including short-course treatments, unless a contraindication exists (*17*,*18*). Health care personnel who do not complete LTBI treatment should be monitored with annual symptom evaluation to detect early evidence of TB disease and to reevaluate the risks and benefits of LTBI treatment. These health care personnel also should be educated about the signs and symptoms of TB disease that should prompt an immediate evaluation between screenings.

Health care facilities should aim to identify LTBI among health care personnel and encourage LTBI treatment. Health care facilities are urged to collaborate with public health agencies to assist in achieving this goal. Public health agencies can serve as a source for technical assistance, medical consultation regarding diagnosis and treatment of LTBI, and clarification of state or local regulations, surveillance requirements, and guidelines. Sharing information and experiences with public health agencies is necessary for understanding the impact of these recommendations on the overall incidence of TB and LTBI in the United States and the need to revise future recommendations for health care personnel.

**Summary**

**What is already known about this topic?**

Since 1991, U.S. tuberculosis (TB) rates have declined, including among health care personnel (HCP). Serial TB testing has limitations in populations at low risk.

**What is added by this report?**

A systematic review found a low percentage of HCP have a positive TB test at baseline and upon serial testing. Updated recommendations for screening and testing HCP include an individual baseline (preplacement) risk assessment, symptom evaluation and testing of persons without prior TB or latent TB infection (LTBI), no routine serial testing in the absence of exposure or ongoing transmission, treatment for HCP diagnosed with LTBI, annual symptom screening for persons with untreated LTBI, and annual TB education of all HCP.

**What are the implications for public health practice?**

Increasing LTBI treatment among HCP might further decrease TB transmission in health care settings.
